# The Identification of a Novel Pathogenic Variant of the *GLA* Gene Associated with a Classic Phenotype of Anderson–Fabry Disease: A Clinical and Molecular Study

**DOI:** 10.3390/ijms26020470

**Published:** 2025-01-08

**Authors:** Irene Giacalone, Luigina Ruzzi, Monia Anania, Mariateresa Cuonzo, Emanuela Maria Marsana, Silvia Mastrippolito, Daniele Francofonte, Silvia Bucco, Annalisa D’Errico, Maria Olimpia Longo, Carmela Zizzo, Luigia Iarlori, Giovanni Duro, Paolo Colomba

**Affiliations:** 1 Institute for Biomedical Research and Innovation (IRIB), National Research Council (CNR), 90146 Palermo, Italy; irene.giacalone@irib.cnr.it (I.G.); monia.anania@irib.cnr.it (M.A.); emanuelamaria.marsana@irib.cnr.it (E.M.M.); daniele.francofonte@irib.cnr.it (D.F.); annalisa.derrico@irib.cnr.it (A.D.); carmela.zizzo@irib.cnr.it (C.Z.); giovanni.duro@irib.cnr.it (G.D.); 2 Nephrology and Dialysis Unit, Hospital “Renzetti”, 66034 Lanciano, Italy; luigina.ruzzi@asl2abruzzo.it (L.R.); mariateresa.cuonzo@asl2abruzzo.it (M.C.); sa.mastrippolito@asl2abruzzo.it (S.M.); silvia.bucco@asl2abruzzo.it (S.B.); molimpia.longo@asl2abruzzo.it (M.O.L.); luigia.iarlori@asl2abruzzo.it (L.I.)

**Keywords:** Fabry disease, *GLA* gene, novel variant, alpha-galactosidase A, c.484delT

## Abstract

Anderson–Fabry (or Fabry) disease is a rare lysosomal storage disorder caused by a functional deficiency of the enzyme alpha-galactosidase A. The partial or total defect of this lysosomal enzyme, which is caused by variants in the *GLA* gene, leads to the accumulation of glycosphingolipids, mainly globotriaosylceramide in the lysosomes of different cell types. The clinical presentation of Fabry disease is multisystemic and can vary depending on the specific genetic variants associated with the disease. To date, more than 1000 different variants have been identified in the human *GLA* gene, including missense and nonsense variants, as well as small and large insertions or deletions. The identification of novel variants in individuals exhibiting symptoms indicative of Fabry disease, expands the molecular comprehension of the *GLA* gene, providing invaluable insights to physicians in the diagnosis of the disease. In this article, we present the case of two members of the same family, mother and son, in whom a new pathogenic variant was identified. This variant has not been previously described in the literature and is not present in databases. The two family members presented with a number of typical clinical manifestations of the disease, including cornea verticillata, neuropathic pain, left ventricular hypertrophy, angiokeratomas and abdominal pain. The son, but not his mother, showed reduced alpha-galactosidase A activity, while high levels of Lyso-Gb3 in the blood, a specific substrate accumulation biomarker, were found in both. Sequencing of the *GLA* gene revealed the presence of a variant, c.484delT, which is characterised by the deletion of a single nucleotide, a thymine, in exon 3 of the gene. This results in a frameshift variant, which introduces a premature stop codon, thereby generating a truncated and consequently non-functional protein. Therefore, the clinical and laboratory data indicate that the novel p.W162Gfs*3 variant described herein is associated with the classical form of Fabry disease.

## 1. Introduction

Anderson–Fabry disease (or Fabry, FD) is a rare metabolic lysosomal storage disorder characterised by a functional defect of the enzyme alpha-galactosidase A (α-gal A) [1]. The functional deficiency of this enzyme is caused by variants in the *GLA* gene, located on the long arm of the X chromosome [2]. This enzyme, a lysosomal hydrolase, is widely expressed in different tissues. Its deficiency results in a progressive accumulation of undegraded glycosphingolipids, mainly globotriaosylceramide (Gb3), within the lysosomes of various cell types, including vascular endothelial cells, cardiomyocytes, podocytes, and cells of the central nervous system [3].

Glycosphingolipids, such as Gb3, are components of the plasma membrane that are subject to degradation within the lysosome by the synergistic action of several hydrolysing enzymes, including alpha-galactosidase A. The accumulation of incompletely degraded substrate affects all cell types and plays a crucial role in the development of Fabry disease. In addition to Gb3, its derivative, globotriaosylsphingosine (Lyso-Gb3), has been identified as a hallmark of FD [4]. Lyso-Gb3 represents the deacylated form of Gb3. The process of converting Gb3 into its deacylated form is catalysed by the lysosomal enzyme acid ceramidase, which facilitates the transformation of Gb3 into a more soluble molecule, thereby enhancing its excretion from cells. This additional catabolic step serves to reduce the cellular accumulation of non-degraded substrate [5]. Lyso-Gb3 is currently the primary metabolite associated with Fabry disease, and its accumulation is a significant contributing factor to the progression of the disease. Recent studies have identified Lyso-Gb3 as a reliable diagnostic marker, and the evaluation of plasma levels of Lyso-Gb3 is a useful tool for determining the severity of the disease in Fabry patients. Observations have revealed that levels of Lyso-Gb3 are elevated in patients with variants that result in a more severe manifestation of the disease, while they are reduced or even normal in late-onset variants or in GVUS (Genetic Variant of Uncertain Significance) [6,7]. Furthermore, the evaluation of Gb3 levels through biopsy and the measurement of circulating Lyso-Gb3 represent a reliable methodology for the monitoring of patient outcomes and the assessment of the efficacy of therapeutic interventions. Indeed, a reduction in Gb3 and Lyso-Gb3 is frequently observed in response to therapy [8,9,10].

The progressive accumulation of Gb3 and Lyso-Gb3 is associated with a wide range of disease-specific signs and symptoms, including renal failure, neuropathies, cardiovascular disorders, stroke, and dermatological manifestations in the form of angiokeratomas [11,12,13]. The clinical manifestations of Fabry disease are characterised by a slow progression, variable onset, severity, and course. Fabry disease manifests in two distinct forms: early-onset (classic phenotype) and late-onset (variable phenotype). The condition affects both males and females, but as it is an X-linked disease, males are generally more severely affected than females [14,15].

Hemizygous male patients with the classic phenotype already present symptoms such as neuropathic pain, hypohidrosis, skin angiokeratomas, gastrointestinal symptoms, corneal opacities, microalbuminuria or proteinuria in early childhood or adolescence. As patients progress through life, the symptoms typically progress to renal failure, cardiac and cerebrovascular involvement, and death between the fourth and fifth decades of life [3,12,14]. Patients with the late-onset phenotype have residual alpha-galactosidase A activity, and the manifestation of symptoms is often delayed and typically involves only a single organ [11,16]. In males affected by the classic form of Fabry, there is very low or no alpha-galactosidase A activity. The diagnosis is made reliably by measuring the enzyme activity in dried blood spots (DBS), plasma, or isolated leukocytes [12]. In affected females who are heterozygous for the disease and present a range of symptoms, from asymptomatic to severe (with involvement of vital organs), the assessment of enzyme activity is less reliable. This is attributable to the random inactivation of one of the two X chromosomes, a process called Lyonization, where a similar or disproportionate number of cells in an organ may preferentially express the active or inactive gene [17]. Consequently, the level of blood Lyso-Gb3 in female patients is similarly unreliable. Therefore, in these patients, genetic analysis is an indispensable and reliable diagnostic tool [18]. Furthermore, the identification of variants in the *GLA* gene can facilitate the comprehension of genotype–phenotype correlations in Fabry patients [19].

Fabry disease is suspected on the basis of clinical data and an accurate family history. Indications for suspecting this condition include cardiac involvement, which is prevalent, albeit with varying degrees of severity, in both male and female patients. The hallmark of this condition is progressive cardiac hypertrophy, fibrosis, arrhythmias, heart failure and sudden cardiac death. The manifestation of left ventricular hypertrophy (LVH) is often the initial clinical indication of Fabry disease, which frequently presents in adulthood. A comprehensive clinical evaluation is performed by a cardiologist, supplemented by instrumental methods such as echocardiography and cardiac MRI. The extent of cardiac damage increases progressively with age and, in conjunction with terminal kidney damage, is the primary cause of death in Fabry disease. The potential for early identification of cardiac involvement prior to the development of left ventricular hypertrophy renders these methods indispensable for the early diagnosis of cardiac damage and, consequently, for the timely initiation of enzyme replacement therapy [20]. The confirmation of Fabry disease is achieved through the implementation of genetic and biochemical tests, which are designed to identify the potential existence of a specific genetic alteration and to assess the activity of the enzyme alpha-galactosidase A. The determination of the enzyme’s substrates, Gb3 and Lyso-Gb3, also contributes significantly to the diagnosis [8].

In this study, we report a novel pathogenic variant, identified through the sequencing of the *GLA* gene, in two members of a family who presented classic clinical manifestations of Fabry disease, reduced enzyme activity and the presence of elevated Lyso-Gb3 in the blood.

## 2. Results

The proband is a 54-year-old woman who, over the course of her life, had undergone several cardiological evaluations for symptoms characterised by heart palpitations, where the electrocardiogram showed “supraventricular tachycardias” classified as anxiety crises, since they arose in conjunction with a family bereavement. The echocardiogram revealed severe, concentric left ventricular hypertrophy and frequent supraventricular extrasystoles. Considering the echocardiographic picture, the patient underwent cardiac MRI (Magnetic Resonance Imaging), which revealed widespread concentric hypertrophy of the left ventricle associated with the presence of more evident areas of LGE (Late Gadolinium Enhancement) in correspondence with the inferior, infero-lateral-basal and middle left ventricular wall, where a focal area of myocardial oedema appeared to be appreciated. These procedures therefore documented cardiac involvement, with lesions typical of Fabry disease [21,22]. Therefore, when the patient came to the attention of the Nephrology and Dialysis department of the Lanciano Hospital, the cardiac damage, in addition to the presence of periumbilical angiokeratomas and the family history, started the evaluation for Fabry disease. In fact, the patient reported that her father died at the age of 42 due to a cerebral stroke and already suffered from severe heart disease, better unspecified, while her 45-year-old sister was alive and had a heart disease in the diagnostic definition phase. Blood tests showed normal renal function (serum creatinine 0.83 mg/dL and GFR 76 mL/min) and the absence of proteinuria. The eye examination revealed cornea verticillata. Brain MRI highlighted findings of an ischemic microvascular nature both in the white matter of frontal subcortical, parietal subcortical, periventricular, and in the cerebellar hemispheres. Furthermore, the ectatic and tortuous dolic appearance of the carotid siphons and slight flow signal of the right vertebral artery were highlighted in the cerebellar hemispheres. The brain MRI showed leukoencephalopathy, and neuropathic pain was probably due to small-fibre neuropathy, typical manifestations of Fabry disease [23] (Table 1).

As indicated by the patient’s clinical and pathological findings, the diagnosis of Fabry disease was suspected. Consequently, a blood sample was sent to the CNR’s Centre for Research and Diagnosis of Lysosomal Accumulation Diseases in Palermo for diagnostic confirmation. The investigation commenced with a genetic analysis, as the patient is female. This is due to the fact that Fabry disease is an X-linked disease, and, due to the phenomenon of lyonization, affected women, who are generally heterozygous for the disease, are a mosaic of normal and diseased cells depending on which of the two X chromosomes has been inactivated in the specific body region. Consequently, enzyme activity values in women exhibit variability, ranging from the normal to the pathological spectrum. This finding underscores the fact that the study of enzyme activity and Lyso-Gb3 accumulation in the blood of female patients is often unreliable and does not serve as a reliable diagnostic tool for the disease.

**Figure 1:**
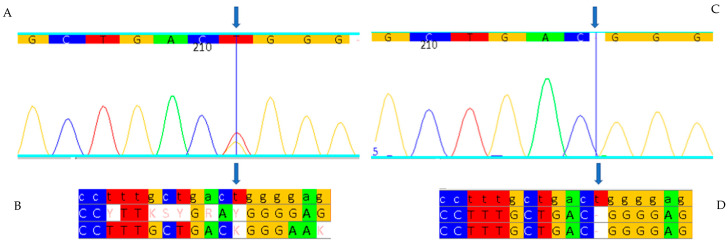
c.484delT pathogenic variant. Analysis of exon 3 of the *GLA* gene in the female patient (**A**) and (**B**) and in the male patient (**C**,**D**). (**A**) Portion of the electropherogram of exon 3 of the *GLA* gene in the female patient in which the c.484delT variant is indicated by the arrow. (**B**) Portion of the sequence of exon 3 of the *GLA* gene in the female patient aligned with the corresponding sequence of a healthy control (wild type). (**C**) Portion of the electropherogram of exon 3 of the *GLA* gene in the male patient in which the c.484delT variant is indicated by the arrow. (**D**) Portion of the sequence of exon 3 of the *GLA* gene in the male patient aligned with the corresponding sequence of a healthy control (wild type). Each base corresponds to a color: red for Thymine, blue for Cytosine, yellow for Guanine, green for Adenine.

In order to ascertain the presence of variants in the *GLA* gene, genomic DNA was extracted from the subject’s peripheral blood, after which the entire coding portion of the gene was amplified using the polymerase chain reaction (PCR). In particular, the seven exons of the *GLA* gene and their flanking regions, which are important for exon splicing, were amplified. In addition, intron 4, which is considered to be a cryptic exon, was amplified. Subsequent to this, the PCR products were analysed using agarose gel electrophoresis, and the amplicons were then subjected to Sanger sequencing. Sequence analysis was then performed using bioinformatics software, with the patient’s sequence then aligned with the wild type reference sequence. The analysis revealed the presence of a heterozygous deletion of a thymine in exon 3 of the *GLA* gene that maps to position 484 of the cDNA. This variant has been described here for the first time and has been designated c.484delT according to the variant nomenclature guidelines recommended by the Human Genome Variation Society (Figure 1). The variant was not identified in the International Fabry Disease Genotype–Phenotype Database (bdFGP) or in other large genetic variant databases, including the Human Gene Mutation Database (HGMD), the ClinVar Database and the Leiden Open Variation Database (LOVD). The pathogenic c.484delT variant causes the amino acid substitution p.W162Gfs*3, the change from a tryptophan to a glycine at position 162 of the protein, as well as a frameshift. In addition to altering the downstream amino acid sequence, the frameshift leads to the formation of a premature stop codon three codons downstream of the variant site, resulting in a truncated and non-functional protein.

**Figure 2:**
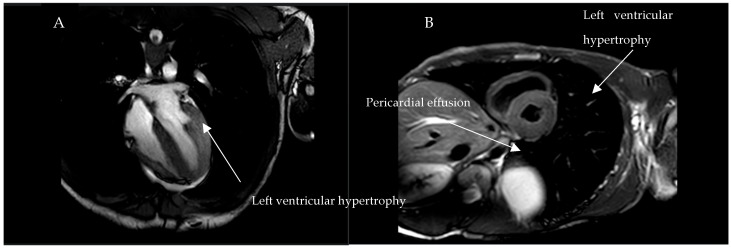
Cardiac MRI of male patient. (**A**) Concentric thickening of the walls of the left ventricle; (**B**) Circumferential pericardial effusion flap.

Following the genetic investigation, biochemical studies were performed using fluorimetric methods to assess the activity of the enzyme alpha-galactosidase A. The patient’s blood level was 5.4 nmol/h/mL (normal levels are above 3 nmol/h/mL). This seems to describe a normal situation, but it must be considered that when analysing the enzyme activity in a woman with FD, it is possible to detect normal values from the pool of normal cells expressing the wild type *GLA* gene; therefore, a correct diagnosis of the disease cannot be made by studying the enzyme activity alone. Once the variant had been identified and the enzyme activity assessed, the presence of Lyso-Gb3 accumulation in the blood was investigated using mass spectrometry. Lyso-Gb3 is the deacylated form of Gb3 and is the diagnostic biomarker for Fabry disease. The level of Lyso-Gb3 in the woman’s blood was 21.76 nmol/L, which is above the normal range of less than 2.3 nmol/L (Table 2). The patient has an only child, a 15-year-old boy, who presented with neuropathic and abdominal pain and was screened for Fabry disease as part of a family history. He reported recurrent abdominal pain in childhood and neuropathic pain manifesting for about a year. The boy’s blood tests showed normal renal function (serum creatinine 0.67 mg/dL and GFR 76 mL/min) and absence of proteinuria (Table 1). Cardiac MRI showed circumferential pericardial effusion and mild concentric hypertrophy of the left ventricle (Figure 2). The ophthalmological examination revealed cornea verticillata. Brain MRI was unremarkable. However, the neurological examination revealed early signs of small fibre neuropathy.

The genetic study also revealed the presence of the hemizygous pathogenic variant c.484delT in the son (Figure 1C,D). The activity of the enzyme alpha-galactosidase A in the blood of the male patient was 0.1 nmol/h/mL (normal values greater than 3 nmol/h/mL). This shows how severely the enzyme is affected and confirms the association of this new variant with the severe phenotype of the disease. The significant blood accumulation of Lyso-Gb3 at 156.50 nmol/L (normal values less than 2.3 nmol/L) also confirms the severity of the variant (Table 2).

Following the diagnosis of Fabry disease, both the mother and the son were expeditiously initiated on enzyme replacement therapy (ERT) on 30 November 2023. The therapeutic regimen entails the administration of 1 mg/kg body weight of the enzyme, administered via intravenous infusion on a biweekly basis. After a period of six months from the commencement of ERT, a reduction in Lyso-Gb3 was observed in both patients when compared to their initial values. In the mother, the level decreased from 21.76 nmol/L to 12.72 nmol/L, while in the son, it decreased from 156.5 nmol/L to 27.48 nmol/L (see Table 3). Furthermore, six months after the commencement of enzyme replacement therapy, the clinical situation of both patients stabilised. Renal function remained normal with the absence of proteinuria in both the mother and son. The study of other relatives of the proband is currently underway, taking into account the X-linked transmission of the disease.

## 3. Discussion

Fabry disease is a frequently encountered but rarely diagnosed condition due to phenotypic variability and multidisciplinary clinical manifestations. In fact, the patient is evaluated by several specialists before receiving a correct diagnosis of the disease. The rarity and complexity of the condition, coupled with the paucity of research in this area, poses significant challenges in attributing clinical and instrumental manifestations to the disease. Consequently, this can result in a diagnostic delay of up to several years, as evidenced in the case study presented here. Over the previous two decades, substantial progress has been made in the diagnosis and treatment of Fabry disease. Given the progressive nature of the condition, it is important to establish an early diagnosis in order to initiate treatment. Indeed, the efficacy of ERT is due to its ability to block and reduce the accumulation of glycosphingolipids at the disease’s target sites [24]. Enzyme replacement therapy has been shown to improve quality of life and reduce the progression of cardiovascular and renal failure in patients with FD [25].

The analysis of variants in the *GLA* gene is an essential step in the correct diagnosis of Fabry disease in individuals with a clinical suspicion of the condition. To date, the Human Gene Mutation Database (www.hgmd.org, accessed on 18 December 2024) has documented over 1000 variants in the *GLA* gene. Nevertheless, a definitive genotype–phenotype correlation remains incomplete [26] and further research is required in this area. A distinctive feature of Fabry disease is the heterogeneity of its clinical manifestations, which complicates its diagnosis. Indeed, considerable variation in clinical symptoms is observed in both hemizygous and heterozygous patients. The phenotypic features are even more variable in female patients (heterozygous for the disease) due to both the nature and type of the *GLA* gene variant and the inactivation profiles of the X chromosome in the different organs. In these patients, the severity of the disease depends on the percentage of cells with the mutated X chromosome that remain active. Thus, in affected women, symptoms may be mild in one individual and severe in another [17]. Due to the heterogeneity of the clinical aspects of Fabry patients, there is a growing interest from the scientific community in establishing potential associations between genotypes and phenotypes in order to optimise the clinical management of this disease.

In this article, we report the case of two members of the same family, mother and son, in whom sequence analysis of the *GLA* gene, compared with the wild type reference sequence, revealed the presence of the c.484delT variant. This is a single-nucleotide deletion, in exon 3 of the *GLA* gene, which causes a shift in the reading frame, with the formation of a premature stop codon. This variant, p.W162Gfs*3, has not previously been described in the literature and is not reported in the Fabry disease-associated variant databases. We describe this variant for the first time and interpret it as pathogenic and responsible for the classic form, as it is a variant that gives rise to a truncated and therefore non-functional protein of 164 amino acids compared to the wild type protein of 429 amino acids. The loss of function of this protein was demonstrated by the low enzyme activity of alpha-galactosidase A found in the male patient, a 15-year-old boy who presented with neuropathic and abdominal pain and left ventricular hypertrophy. The mother’s enzyme activity level, on the other hand, was normal. This is not surprising as the level of enzyme activity in women can vary from normal to low. This is due to the phenomenon of lyonization, the random inactivation of one of the two X chromosomes, which makes women with Fabry disease a mosaic of normal and affected cells. Therefore, in a given tissue, depending on which X chromosome is silenced (the mutant or the wild type), the protein produced will be a functional or inactive enzyme. In women, the severity of the disease depends on the percentage of cells with the mutated X chromosome that remain active. This results in considerable clinical heterogeneity, ranging from no manifestations to a severity comparable to that of males with Fabry disease [27,28,29]. The study of enzyme alterations therefore presents different aspects in male and female patients due to lyonization, and it follows that in females it is necessary to proceed directly to genetic analysis, as their enzyme activity is not indicative in the diagnosis of Fabry disease [18]. With regard to the assessment of lyso-Gb3 accumulation in the blood, we found high levels in women and very high levels in children. It has been shown that blood accumulation levels of Lyso-Gb3 are significantly higher in subjects with variants associated with a classic phenotype, such as frameshift or nonsense variants responsible for the formation of stop codons during the translation phase, or variants that fall within splicing sites, where control of gene expression occurs at the post-transcriptional level, whereas, Lyso-Gb3 levels are more modest in subjects with a late-onset phenotype [7].

After ERT, patients showed a decrease in LysoGb3 accumulation and clinical symptoms stabilised.

Considering the patients’ symptoms, the alpha-galactosidase A enzyme activity values in the male patient, the accumulation of Lyso-Gb3 in the blood of both patients, and the nature of the genetic alteration itself, the c.484delT variant can be considered causative of the classic form of Fabry disease.

## 4. Materials and Methods

Patients’ peripheral blood was collected, using EDTA as an anticoagulant, and dried on absorbent paper (dried blood spot, DBS). Genetic and enzymatic studies were performed at the Center for Research and Diagnosis of Lysosomal Storage Disorders of IRIB-CNR in Palermo.

### 4.1. α-Galactosidase a Activity Assay

α-galactosidase A activity assays were performed using the dried-blood filter paper (DBFP) test described by Chamoles et al. [30], with some modifications. A spot of 10 µL of blood in a circle of paper 6 mm in diameter was placed into a 96-well plate, suitable for fluorometric assays, and incubated for 18 h at 37 °C in a thermomixer; the reaction was terminated by the addition of 250 µL of 0.1 mol/L ethylenediamine (pH 11.4). The background fluorescence, i.e., fluorescence which was not due to the specific enzyme activity, was determined for each sample, conducting another reaction in the presence of 0.14 mmol/L 1-deoxygalactonojirimycin (DGJ, the inhibitor of alpha galactosidase A) in citrate phosphate buffer (pH 4.5). This background was subtracted from the fluorescence of the sample. In each assay, we added positive and negative controls and a calibration curve with 4-methylumbelliferone. Normal values were >3.0 nmol/h/mL.

### 4.2. Genetic Analysis

Genomic DNA was isolated from a dried blood spot using silica-coated magnetic particles in a robotic workstation designed for automated purification of nucleic acids. DNA concentrations were estimated using a biophotometer (Eppendorf, Hamburg, Germany). The search for variants in the *GLA* gene was performed using Sanger sequencing. Eight pairs of primers were designed to analyse eight target regions containing the seven exons of the *GLA* gene, including the flanking regulatory sequences, and the cryptic exon. PCR products were purified and sequenced at Eurofins Genomics (Ebersberg, Germany).

### 4.3. LysoGb3 Determination

The determination of LysoGb3 in blood was performed via tandem mass spectrometry (MS/MS) methodology, as previously described by Polo et al. [31].

## 5. Conclusions

Fabry disease is still difficult to diagnose, mainly because its clinical manifestations overlap with those of other diseases. In fact, patients often have to see several specialists before the correct diagnosis is made. Diagnostic error is therefore a real risk, not only leading to an underestimation of the true number of sufferers, but also preventing the possibility of timely therapeutic treatment that can significantly improve symptoms and slow the progression of the disease. It also limits the possibility of extending genetic testing to family members once the proband has been identified, thus preventing the early diagnosis of other affected family members and the timely administration of therapy. The correct diagnosis of the disease is also severely hampered by the phenotypic variability that occurs between both related and unrelated patients. Indeed, phenotypic heterogeneity associated with the same variant is often a factor that hinders the study of genotype–phenotype correlation in Fabry disease. The results reported here provide substantial evidence that the novel c.484delT variant in the *GLA* gene is causative of the classic form of Fabry disease in the two patients who presented with reduced enzyme activity, accumulation of Lyso-Gb3 and classic signs of the disease. These novel findings may assist in the diagnosis of Fabry disease and enhance the clinical and molecular understanding of the correlations between variants in the *GLA* gene and the Fabry phenotype.

## Figures and Tables

**Table 1:** Clinical data of the two patients. The + symbol indicates the presence of the sign/symptom, while the − symbol indicates the absence of the sign/symptom.

Patient	Sex/Age	Neuropathic Pain	Cornea Verticillata	Left Ventricular Hypertrophy	Cardiac Manifestations	Renal Manifestations	Neurological Manifestations	Other Signs
1	F/54	+	+	+	+	−	+	Periumbilical angiokeratomas
2	M/15	+	+	−	+	−	+	Abdominal muscle pains

**Table 2:** The pathogenic variant causing Fabry disease in the examined patients.

Patient	Sex	Age	Mutation	Alpha-Galactosidase A Activity (nmol/h/mL) Normal Range: >3.0	Lyso-Gb3 in Blood (nmol/L) Normal Range: >3.0
1	F	54	c.484delT heterozygote	5.4	21.76
2	M	15	c.484delT hemizygote	0.1	156.50

**Table 3:** Changes in Lyso-Gb3 accumulation levels before and after the start of therapy.

Blood Lyso-Gb3 Accumulation Value				
Patient	Sex	Age	Before the Start of Therapy	Six Months After the Start of Therapy
1	F	54	21.76 nmol/L	12.72 nmol/L
2	M	15	156.50 nmol/L	27.48 nmol/L

## Data Availability

Data are contained within the article.
